# A Promiscuous DNA Packaging Machine from Bacteriophage T4

**DOI:** 10.1371/journal.pbio.1000592

**Published:** 2011-02-15

**Authors:** Zhihong Zhang, Vishal I. Kottadiel, Reza Vafabakhsh, Li Dai, Yann R. Chemla, Taekjip Ha, Venigalla B. Rao

**Affiliations:** 1 Department of Biology, The Catholic University of America, Washington, District of Columbia, United States of America; 2 Department of Physics, University of Illinois at Urbana-Champaign, Urbana, Illinois, United States of America; 3 Howard Hughes Medical Institute, Urbana, Illinois, United States of America; Princeton University, United States of America

## Abstract

Complex viruses are assembled from simple protein subunits by sequential and irreversible assembly. During genome packaging in bacteriophages, a powerful molecular motor assembles at the special portal vertex of an empty prohead to initiate packaging. The capsid expands after about 10%–25% of the genome is packaged. When the head is full, the motor cuts the concatemeric DNA and dissociates from the head. Conformational changes, particularly in the portal, are thought to drive these sequential transitions. We found that the phage T4 packaging machine is highly promiscuous, translocating DNA into finished phage heads as well as into proheads. Optical tweezers experiments show that single motors can force exogenous DNA into phage heads at the same rate as into proheads. Single molecule fluorescence measurements demonstrate that phage heads undergo repeated initiations, packaging multiple DNA molecules into the same head. These results suggest that the phage DNA packaging machine has unusual conformational plasticity, powering DNA into an apparently passive capsid receptacle, including the highly stable virus shell, until it is full. These features probably led to the evolution of viral genomes that fit capsid volume, a strikingly common phenomenon in double-stranded DNA viruses, and will potentially allow design of a novel class of nanocapsid delivery vehicles.

## Abstract 2

Packaged viral genome can be removed from bacteriophage T4 capsid and the capsid refilled with any double-stranded DNA, single or multiple molecules, using a powerful ATP-fueled DNA packaging machine.

## Author Summary

Phage T4 is a prototype for tailed bacteriophages, the most abundant organisms on the planet, as well as for large eukaryotic viruses such as herpes viruses. These viruses encode powerful machines to package their genomes tightly inside an icosahedral-shaped capsid “head.” Packaging into the capsid occurs via a dodecameric portal, localized in one of the vertexes of the capsid. Packaging requires precise orchestration of a series of steps: assembly of an empty prohead, concatemer cutting and attachment of the motor-DNA complex to the portal vertex, ATP-fueled DNA translocation until the head is full, DNA cutting to terminate packaging, detachment of the motor, and sealing of the packaged head by “neck” assembly. Sequential conformational changes, particularly in the portal, are thought to drive these transitions such that assembly proceeds directionally and irreversibly. Here, we found that the phage T4 packaging machine on various capsids is highly promiscuous, translocating DNA into proheads but also, unexpectedly, into previously filled virus heads. Other studies have shown that in filled viral capsids the structure of the portal is fundamentally altered, and it was thought that the packaging mechanism on full heads would be irreversible. We show that full heads, or heads that are emptied of most of their packaged DNA, can reassemble the packaging machine and use it to re-fill the capsid with any DNA molecules. These results challenge the classic sequential virus assembly models, suggest an explanation for the evolution of viral genomes that fit capsid volume, and point the way to a novel nanocapsid delivery system in which the viral packaging machine (portal and motor) could be used to translocate DNA and other therapeutic molecules into synthetic capsids.

## Introduction

Tailed bacteriophages are ubiquitously distributed in nature and are the most abundant organisms on the planet [1]. These, in particular, bacteriophage T4, are excellent models to elucidate the mechanisms of DNA condensation and decondensation in living organisms. The virion consists of a head into which the genome is packaged, and a tail that delivers the genome into the bacterial cell. The head is pressurized to ∼6 MPa—equivalent to more than ten times the pressure inside a bottle of champagne—because of the packing of highly negatively charged, relatively rigid double-stranded DNA (dsDNA) to near crystalline density (∼500 µg/ml) [2],[3].

**Figure 1:**
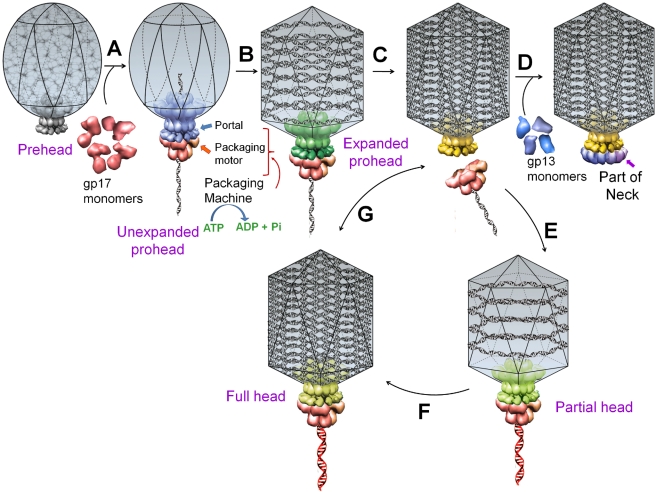
A schematic of DNA packaging by sequential assembly and promiscuous assembly. The major capsid protein assembles around a scaffolding core into a prehead. The core is removed by proteolysis to produce an empty unexpanded prohead (A). The unexpanded prohead normally has a round shape, but in phage T4 it has angular geometry [45]. The packaging motor–DNA complex docks on portal and initiates packaging. The prohead expands after about 10%–25% of the DNA is packaged (B). After headful packaging, the motor cuts the concatemeric DNA and dissociates from the DNA-full head (C). The neck proteins (gp13, gp14, and gp15) assemble on portal to seal off the DNA-full head and provide a platform for tail assembly (D). The various colors of portal represent different conformational states. In promiscuous assembly, the packaging motor assembles on a partial head produced by ejection of packaged DNA (E) or a full head (G), and refills the head with new fragments of DNA ([F] and [G]; new DNA fragments shown in red).

Common pathways and mechanisms are involved in building dsDNA viruses [4]–[6]. A capsid of precise dimensions is first assembled, often with a single type of protein subunit polymerizing around a protein scaffold (Figure 1). A cone-shaped dodecameric portal initiates assembly and remains at the special five-fold vertex of the isometric capsid (prehead), facilitating all subsequent transactions: DNA entry, tail attachment, and DNA ejection [7],[8]. The scaffold is removed, creating an empty space inside the capsid (prohead or procapsid) for encapsidating the viral genome (Figure 1A). A packaging ATPase motor, also known as the “terminase,” recognizes and cuts the concatemeric viral DNA and docks at the narrow protruding end of the prohead portal, inserting the DNA end into the ∼3.5-nm portal channel [9]. The packaging machine thus assembled drives DNA translocation utilizing the free energy of ATP hydrolysis (Figure 1B). After filling the head (“headful” packaging), the motor cuts the DNA and dissociates from the DNA-full head (Figure 1C) [10]. The neck and tail proteins assemble on the portal, completing the infectious virus assembly (Figure 1D) [11]–[15].

The phage T4 packaging motor is the fastest and most powerful reported to date [16]. It generates ∼60 pN of force and packages at a rate of up to ∼2,000 bp/s. The motor is composed of a large terminase protein, gp17 (70 kDa), and a small terminase protein, gp16 (18 kDa) [17]. gp17 contains all the enzymatic activities necessary for DNA packaging: ATPase, nuclease, and translocase [18]–[20]. Five molecules of gp17 assemble on the portal, forming a pentameric motor with a central translocation channel that is continuous with the portal channel [21]. gp16, a putative 11-mer [22], regulates gp17′s activities, but its location on the packaging machine is unknown [23]. Structural and biochemical studies suggest that packaging is driven by the electrostatic force generated by the motor alternating between relaxed and tensed conformational states [21].

A fundamental feature of virus assembly is “sequential assembly” in which “simple” components assemble in a strict sequence to generate a complex nanomachine with unique biological properties. Each assembly step generates a new site or conformational state to which the next component binds with exquisite specificity, essentially irreversibly [24]. A series of such steps, as documented by elegant studies in phage T4 [25] and numerous other viruses [4], leads to rapid and high-fidelity assembly of a complex infectious virion. In phage T4, this process assembles virions approaching a theoretical infection efficiency of 1.

The sequence of steps in the head morphogenesis of phage T4 (in vivo), as well as in other phages and dsDNA viruses (e.g., herpes viruses), is as follows: (i) assembly of the packaging motor on a nascent (unexpanded) empty prohead (Figure 1A), (ii) expansion of the capsid after about 10%–25% of the genome is packaged (Figure 1B), (iii) packaging until the head is full, (iv) cutting of DNA and dissociation of the motor (Figure 1C), and (v) assembly of neck proteins to seal the packaged heads (Figure 1D). Conformational changes in the portal are reported to drive these sequential irreversible transitions (Figure 1; different colors of portal represent different conformational states) [11]–[14].

Here, we report that the assembly of the phage T4 genome packaging machine does not strictly adhere to the above paradigm (“motor” refers to pentameric gp17 whereas “machine” refers to the complete packaging unit including shell [gp23], portal, and motor). Our results show that the assembly of the phage T4 packaging machine is highly promiscuous and does not discriminate the type of head it assembles on. First, we demonstrate that the motor can translocate into the phage head, either the DNA-full head (Figure 1G) or the once full but DNA-ejected head (Figure 1F). In fact, the latter shows 5- to 10-fold greater packaging efficiency than the prohead. This is the first report to our knowledge demonstrating that a finished virus shell can reassemble the packaging machine and repackage any DNA. Second, single molecule optical tweezers experiments show that the packaging rate of phage-head-assembled packaging machines is similar to that of machines assembled on packaging-naïve proheads. Third, single molecule fluorescence measurements show that the mature phage heads catalyze repeated packaging initiations, encapsidating multiple DNA molecules within the same head. These results suggest that the phage T4 DNA packaging machine has unusual conformational plasticity, powering genome translocation into a passive capsid receptacle regardless of its maturation stage. These features might have driven the evolution of headful measured genomes in dsDNA viruses, and may offer avenues to design novel nanodevices that can transport DNA therapeutics and vaccines into cells.

## Results

### Phage Heads Can Reassemble a Functional DNA Packaging Machine and Package DNA

Phage T4 gp10 in association with gp11 forms the tail-pins of the baseplate [26]. Since the tail-pin assembly is the first step of tail assembly, tail structures do not assemble in the absence of gp10. The proteins gp13, gp14, and gp15 assemble into a neck that seals off packaged heads, with the gp13 protein directly interacting with the portal protein gp20 following DNA packaging and gp14 and gp15 then assembling on the gp13 platform. It has been well documented that *10am13am* mutants (and analogous mutants in phage λ and other phages) complete all the packaging steps including the cutting of concatemeric DNA and dissociation of the packaging motor ([5],[11] and references therein, [13],[14]). DNA-full phage heads accumulate in the *10am13am* mutant infected cells, which can be converted to infectious virions by in vitro complementation with neck and tail proteins [15]. Thus, according to the well-accepted sequential assembly models, the heads following completion of DNA packaging are expected to have the least affinity for the packaging motor but high affinity for the neck proteins. Surprisingly, our data demonstrate that the packaging machine does not discriminate between “prohead” (Figure 1, step A) and finished or matured “phage head” (steps F and G).

**Figure 2:**
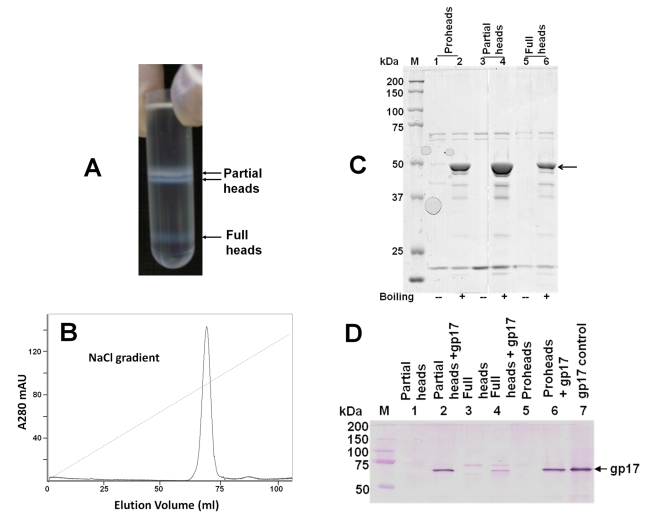
Purification and characterization of phage heads. (A) The *10am13am* heads were isolated by differential centrifugation followed by CsCl gradient centrifugation (see Materials and Methods for details). The two closely spaced bands at the top of the gradient contained partial heads that had ejected most of their packaged DNA, save an ∼8-kb piece. The band at the bottom of the gradient contained full heads in which the packaged T4 genome was stabilized (see Figure 3 legend for additional details). (B) Purification of partial heads by DEAE-Sepharose column chromatography. The two closely spaced head bands at the top of the CsCl gradient were pooled, dialyzed against 10 mM Tris-HCl (pH 7.5), 50 mM NaCl, and 5 mM MgCl_2_, and purified by ion-exchange chromatography (AKTA Prime, GE Healthcare). The column was pre-equilibrated with 50 mM Tris-HCl (pH 7.5) and 5 mM MgCl_2_, and a linear gradient of 0–300 mM NaCl was applied to elute the bound heads. The peak fractions were pooled, concentrated by filtration, and stored at 4°C. (C) The partial and full heads are fully expanded. The purified proheads, partial heads, and full heads were mixed with SDS gel loading buffer and kept at room temperature (“−”) or boiling temperature (“+”) for 5 min. The samples were then separated by 10% SDS-PAGE, stained with Coomassie blue R, and destained. Note that the major capsid protein, gp23* (position marked with arrow), was not seen in the room temperature samples because the expanded heads could not be dissociated into gp23* subunits. (D) Partial and full heads reassemble with the exogenous gp17. About 5×10^11^ proheads, partial heads, or full heads were incubated with purified gp17-K577 (0.3 µM; 50:1 ratio of gp17 molecules to gp20 subunits) in a buffer containing 50 mM Tris-HCl (pH 7.5), 100 mM NaCl, and 5 mM MgCl_2_, at room temperature for 30 min. The head-gp17 complexes were sedimented by centrifugation at 18,000 rpm for 45 min, and the pellet was washed several times to remove any unbound gp17. The proteins were transferred to PVDF membrane, and Western blotting was performed using polyclonal gp17 antibodies. The results were confirmed by doing the same experiment with full-length gp17 and a GFP-gp17 fusion protein. Only the gp17-K577 (C-terminal 33 amino acids of gp17 were deleted) data are shown because gp17-K577 is protease resistant and migrates as a single band as opposed to three bands with the full-length gp17 and GFP-gp17, and also because there is no background overlapping band at the same position. The gp17 band in the full head lane (lane 4) is faint because some of these heads released the packaged DNA during the procedure, which resulted in poor recovery of the heads during the centrifugation and washing steps.

**Figure 3:**
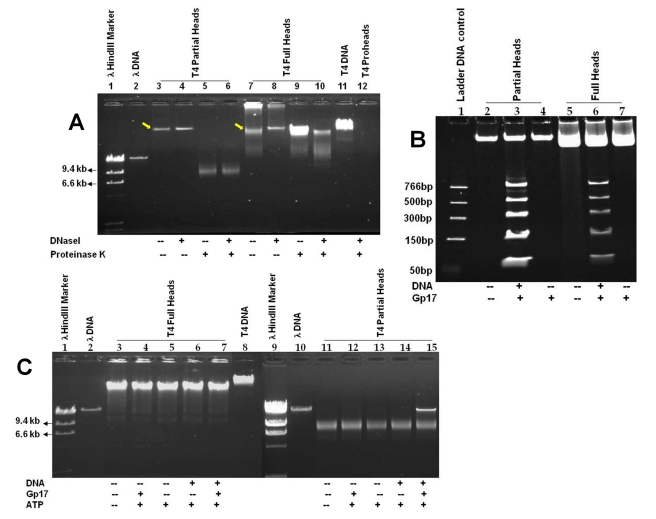
The T4 packaging machine repackages DNA into phage heads. (A) Partial heads (lanes 3–6), full heads (lanes 7–10), or proheads (lane 12) were treated with DNAse I (37°C, 30 min) and/or proteinase K (65°C, 30 min), as shown by “+” or “−” rows under the figure, and subjected to agarose gel (0.8% w/v) electrophoresis and stained with cyber green. The molecular size markers λ HindIII (lane 1), λ DNA (lane 2), and T4 DNA (lane 11) were used to determine the size of the DNA present in the heads. Partial head lanes 3–6: lane 3, without any treatment; lane 4, treated with DNAse I; lane 5, treated with proteinase K; lane 6, treated first with DNAse I and then with proteinase K. Arrow shows the position of the heads stained with cyber green because they were associated with a ∼8-kb DNA (lane 3). The 8-kb DNA was inside the heads because it was resistant to DNAse I treatment (lane 4) but released by treatment with proteinase K (lanes 5 and 6). Full head lanes 7–10: lane 7, without any treatment; lane 8, treated with DNAse I; lane 9, treated with proteinase K; lane 10, treated first with DNAse I and then with proteinase K. Note that the untreated full heads showed, in addition to the head band (arrow), an intensely stained band in the well plus a smear (lane 7), both of which were removed by digestion with DNAse I (lane 8). This was because some of the full heads extruded the packaged DNA during storage, which remained complexed with the head and retained in the well. This was confirmed by treatment with proteinase K, which released this DNA as well as that packaged inside, producing a single band (lane 9). Treatment first with DNAse I resulted in the digestion of the outside DNA, and subsequent addition of proteinase K digested the capsids and released the DNA packaged inside (lane 10). The DNA in lanes 9 and 10 is slightly shorter than that isolated from phage (lane 11), presumably because a segment of packaged DNA near the portal was accessible to DNAse I digestion [11],[13],[14]. Arrow shows the position of the heads stained with cyber green because they were associated with DNA inside the head (lane 7). The control *17am18amrII* proheads are empty and showed no staining with cyber green (lane 12). (B and C) Packaging of short DNA fragments (B) (50–766 bp), or λ DNA (C) (48.5 kb) under various reaction conditions, as shown under the figure. See Materials and Methods for additional details.

The *10am13am* heads were separated into two major species by CsCl density gradient centrifugation (DNA sequencing showed that the *10am13am* phage has TAG amber mutations at residues Trp 430 in gene 10 and Gln 39 in gene 13). Two very closely spaced low-density bands were present at about the middle of the gradient, and a high-density band was located near the bottom of the gradient (Figure 2A). The two close bands, making about 93% of the total heads, contained the same head species but migrated slightly differently, probably because the heads in the upper band were loosely associated with cell debris (in some purifications, only a broad single band was seen). Upon further purification by diethylaminoethyl cellulose (DEAE) ion-exchange chromatography, the cell debris contaminants were removed and the heads eluted as a single symmetrical peak (Figure 2B). Both the head species were resistant to SDS at room temperature (Figure 2C), which means that they are, as expected, in the fully expanded state [27]. Agarose gel electrophoresis showed that the low-density heads contained a ∼8-kb DNA band (Figure 3A, lanes 5 and 6) whereas the high-density heads contained near genome length DNA (Figure 3A, lanes 9 and 10; see figure legend for details). The former are referred to as “partial” heads and the latter as “full” heads.

Since *13am* mutants accumulate DNA-full heads [15], the partial heads likely arose by spontaneous ejection of the packaged DNA from full heads during the purification procedures. The full heads are known to be unstable and to spontaneously eject the DNA unless sealed off by neck proteins [11],[14]. The ejected DNA would be digested by the DNAse I present in the buffer, leaving only a small piece of DNA inside the shell. As seen in Figure 3A, the DNA associated with the partial heads was inside the head because it comigrated with the head band (lane 3) and was resistant to DNAse I treatment (lane 4), but upon digestion with proteinase K, the DNA was released and migrated to the 8-kb position (lane 5). It is interesting that the 8-kb band is consistently observed in several independent preparations and is quite compact, suggesting that ejection stopped within a narrow window, after about 95% of the genome had been released. It can be argued that this DNA belongs to a specific sequence of the T4 genome because it bound to the capsid protein and was not ejected. To test this hypothesis, the DNA was extracted from partial heads by phenol and chloroform and digested with the restriction enzymes EcoRV (six-base cutter) or TaqI (four-base cutter), which can cut the hydroxymethylated and glycosylated T4 DNA. If the 8-kb DNA belongs to a unique sequence of T4 genome, a series of discrete bands should result. Alternatively, if each 8-kb piece belongs to a different part of the genome, the restriction fragments should not form discrete bands. The results showed that the products migrated as a smear (data not shown), demonstrating that the retained DNA does not have a unique sequence. This result is also consistent with the fact that the ends of the T4 genome are nearly random, and thus it is not expected that the stretch of the genome that is in proximity to the capsid protein will be of the same DNA in different particles [28].

The full heads, which make up to about 7% of the total heads, have the packaged genome relatively stably retained inside the head, presumably because either the portal channel was constrained [3] or the DNA ends were not in close proximity to the entrance of the portal channel. However, we found that these heads slowly released DNA upon storage at 4°C.

The packaging activity of partial and full heads was determined by the defined in vitro DNA packaging assay, using the *17am18amrII* empty proheads as a positive control. In phage T4, the empty proheads produced by packaging-defective *17am* mutant infections are mostly of the expanded type (see lanes 1 and 2 of Figure 2C) since expansion occurs spontaneously when packaging is blocked in vivo. The resultant packaging-naïve empty expanded proheads, which package DNA as well or better than the unexpanded proheads, have been used as a positive control in the packaging assays [27],[29],[30]. In bulk packaging assays, both partial and full heads efficiently packaged short DNA fragments (50–766 bp) (Figure 3B). In fact, we found that the packaging efficiency of partial heads was about six times higher than that of the proheads, the true precursors of DNA packaging in vivo (bulk packaging data of proheads not shown; packaging efficiency is defined as the number of DNA molecules packaged per number of head particles). This was likely because the proheads, unlike the mature heads, are fragile and might have been damaged during purification because of irregular expansion in vitro and/or lack of the stabilizing capsid decoration proteins Soc and Hoc [31]. The efficiency of partial head packaging was about five to ten times higher than that of the full heads (Figure 3B, lanes 3 and 6), which is not surprising because most of the full heads may not have any empty space left to accommodate additional DNA. Consistent with this hypothesis, the partial heads, but not the full heads, packaged the 48.5-kb phage λ DNA (Figure 3C, lanes 7 and 15) or the T4 genomic DNA (data not shown). To confirm that the partial and full heads reassembled the exogenously added packaging motor, the head-gp17 complexes were purified and analyzed by Western blotting using polyclonal gp17 antibodies. The data showed that both types of heads reassembled the externally added gp17 (Figure 2D). The above findings were reproduced by constructing additional *10am13am* phage mutants in which either *hoc* or *soc*, or both genes, were also deleted (data not shown).

### Full Heads Can Package DNA

**Figure 4:**
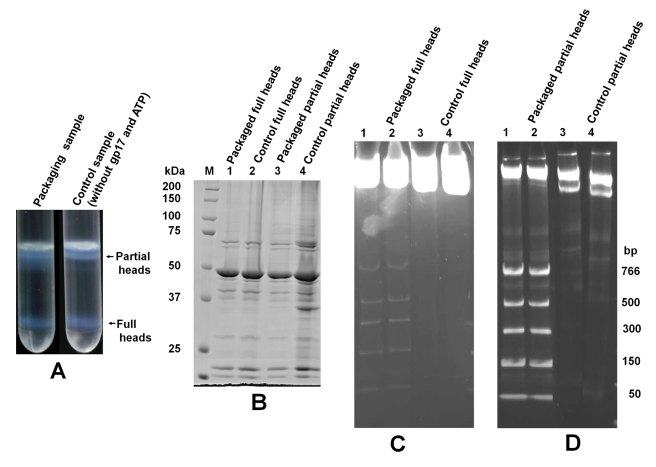
Full heads can package DNA. (A) The phage heads were isolated from *10am13am* infected *E. coli* P301 cells (500 ml culture) by lysis in the presence of DNAse I followed by differential centrifugation (see Materials and Methods for details). The phage head pellet containing a mixture of partial heads and full heads was resuspended in 200 µl of 50 mM Tris-HCl (pH 7.5) and 5 mM MgCl_2_. The sample was split into two halves, and larger scale packaging assays were conducted immediately. The 500-µl packaging reactions contained 100 µl of phage heads, 4.75 µM GFP-gp17, 43 µg of ladder DNA (50–766 bp; NEB), 5% PEG buffer, and 1 mM ATP [30]. gp17 and ATP were omitted in the control reaction. After 30 min of incubation at room temperature, 40 µl (1,000 units) of Benzonase nuclease (EMD Biosciences) was added to digest unpackaged DNA, and the samples were separated by CsCl density gradient centrifugation. (B) The partial and full head samples from the gradient were electrophoresed on 10% SDS polyacrylamide gel to analyze for proteins and to estimate the concentration of particles used in the packaging reactions. Since the concentration of full heads is very low compared to that of partial heads (roughly 1/10^th^ that of partial heads), the full heads were concentrated by high-speed centrifugation such that the number of particles per lane are approximately the same for both full heads and partial heads. (C and D) The full (C) and partial (D) head bands from the gradient were treated with proteinase K (18.5 µg; Fermentas) and electrophoresed on 4%–20% polyacrylamide gel in Tris-borate buffer (pH 8) to analyze for packaged DNA.

Although the above results show that full heads package DNA, it can be argued that a fraction of the full heads ejected DNA during CsCl gradient centrifugation, converting them into partial heads. To address this question, *10am13am* heads were prepared without the CsCl gradient centrifugation. The infected cells were lysed in the presence of DNAse I, and phage heads were isolated by differential centrifugation. These heads, which contained a mixture of partial heads and full heads, were packaged with DNA (50- to 766-bp ladder fragments) and then separated by CsCl density gradient centrifugation (see Materials and Methods and Figure 4 legend for details). This not only minimized any DNA ejection from full heads but, more importantly, ensured that only the full heads that packaged DNA sedimented to the high-density position (lower band) in the CsCl gradient.

The partial and full head bands (Figure 4A) were extracted from the CsCl gradient, treated with proteinase K to release packaged DNA, and electrophoresed on a 4%–20% polyacrylamide gel. The samples were also electrophoresed prior to proteinase K treatment on an SDS polyacrylamide gel to determine the number of head particles (Figure 4B). As shown in Figure 4C, the full heads packaged the ladder DNA fragments at similar efficiency as the gradient-purified full heads shown in Figure 3 did. Control samples in which the heads were treated the same way except that gp17 and ATP were omitted in the packaging reactions showed no detectable DNA (Figure 4C, compare packaged lanes 1 and 2 to control lanes 3 and 4). The partial heads, as expected, also packaged DNA at a similar efficiency as the gradient-purified partial heads (Figure 3). Indeed, the packaged partial head band showed a downward shift towards higher density after packaging (see the packaged left gradient tube in Figure 4A showing broadening of partial head band towards higher density when compared to the control gradient tube on the right). These experiments suggest that there are no fundamental barriers to packaging short pieces of DNA into full heads, an observation further confirmed by single molecule optical tweezers experiments (see below).

### Single Mature-Phage-Head-Assembled Packaging Machines Refill the Capsid

**Figure 5:**
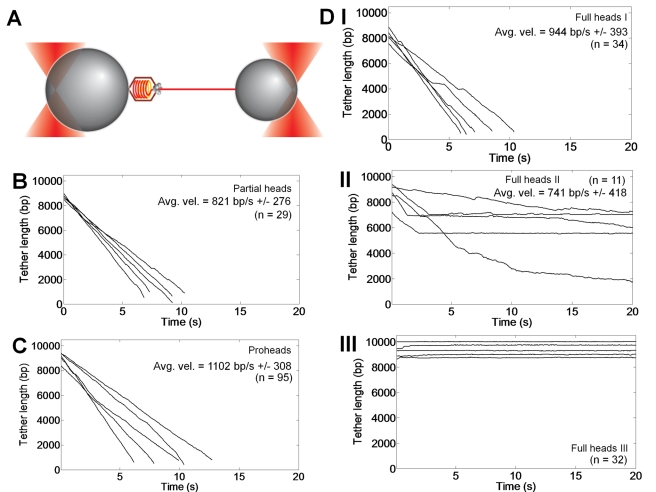
Single mature-phage-head-assembled packaging machines refill the capsid. (A) The dual optical trap set-up for single molecule DNA packaging. The T4 head–motor complex and the 10-kb DNA substrate were tethered between two beads, each held in an optical trap and held under 5 pN tension. See Materials and Methods for details. (B–D) Packaging traces showing the packaging of DNA by proheads (B), partial heads (C), and full heads (DI–DIII). “n” represents the number of packaging traces qualitatively showing similar packaging behavior in that panel.

Single molecule experiments were conducted using dual-trap optical tweezers in a “force–clamp” mode. Head-gp17 packaging complexes were formed in the presence of the non-hydrolyzable analog, ATP-γ-S, and immobilized on T4-antibody-coated microspheres. The substrate DNA molecules (10 kb) biotinylated at one end were attached to streptavidin-coated microspheres. The microspheres were captured in separate traps and brought into near contact and quickly separated (Figure 5A). This “fishing” procedure was repeated until a tether was formed, as evident by a rise in force when the motor captured the DNA. A constant force of 5 pN was then applied by a feedback loop, and packaging was measured as decrease in tether length as a function of time.

The data showed that the packaging rates of the partial-head-assembled packaging machines (Figure 5B) are similar to those of the empty proheads (Figure 5C) (∼800–1,100 bp/s). As reported previously [12], these rates are about seven times faster than those of the phi29 packaging machine. The full heads also packaged DNA but showed distinctive features (Figure 5D). Some of the heads packaged the entire 10-kb DNA, and the packaging rates are similar to those of the partial-head- or prohead-assembled machines (Figure 5DI). These heads presumably emptied a significant portion of the packaged DNA during storage, creating room to accommodate a 10-kb piece. A second class of heads packaged only a short piece of DNA, about 1–3 kb, and then stalled, suggesting that these heads may be nearly full and can only accommodate a small piece (Figure 5DII). Interestingly, the packaging rates of these machines are still very high considering that these machines presumably packaged into a nearly full head. However, some of these machines did not stall completely but instead packaged slowly (e.g., the top and bottom traces in Figure 5DII). A third class of heads simply formed tethers, with no translocation evident, suggesting that these heads may have had no room left to accommodate additional DNA (Figure 5DIII). It is interesting that these machines formed tethers, an indication that they successfully initiated packaging (as evident by a rise in force) but remained in the stalled state for a long period of time (otherwise, the DNA would have rapidly slipped out under 5 pN of force). These data demonstrate that the packaging machines can efficiently assemble on mature phage heads and refill the capsid, and that the length of refilled DNA appears to be dependent upon the amount of space available inside the capsid.

### The Mature-Phage-Head-Assembled Packaging Machines Undergo Multiple Packaging Initiations

**Figure 6:**
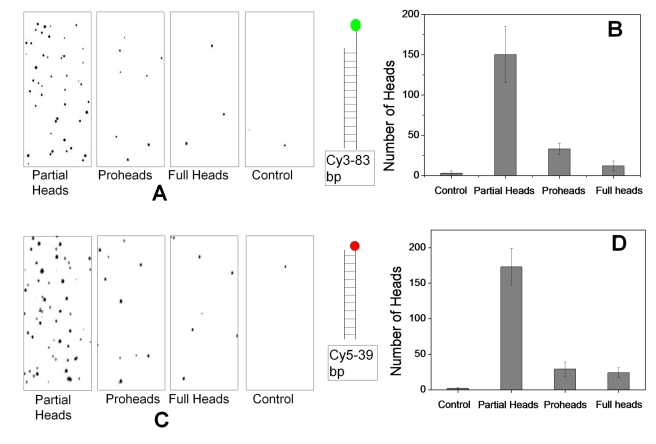
Single molecule fluorescence measurements of refilled heads. Quantification of packaging by single molecule fluorescence assay. See Materials and Methods for details. (A and C) Fluorescence images of immobilized T4 heads packaged with Cy3 (83-bp) and Cy5 (39-bp) DNAs, respectively. One-fourth of the 70 µm ×35 µm imaging area is shown in each case (see Figures S1 and S2 for full-size fluorescent images). (B and D) Histograms showing the number of heads packaged with Cy3 or Cy5 DNAs. The number of heads showing fluorescence in more than 30 images was averaged in each case.

Short 39-bp Cy5-end-labeled and 83-bp Cy3-end-labeled DNAs were packaged into proheads, partial heads, and full heads using the bulk assay. The packaged heads were immobilized on polyethylene glycol (PEG)–passivated quartz surface using anti-phage-T4 polyclonal antibodies, and total internal reflection microscopy and single molecule detection were used to image the fluorescent particles. The “glowing” heads were quantified by determining the average number of bright spots per area from at least 30 images per sample (Figure 6A and 6C; see Figures S1 and S2 for fluorescent images). Consistent with the bulk assays, the average number of bright spots corresponding to partial heads that had packaged the labeled DNA was about 5-fold greater than for the empty proheads, and about 10-fold greater than for the full heads (Figure 6B and 6D). Control experiments, which omitted gp17, had 0–2 bright spots, suggesting that nonspecific fluorescence of any surface-bound material is negligible (the packaged samples were treated with excess DNAse I [10 µg/ml] at room temperature for about 20 h to digest any unpackaged or nonspecifically bound DNA; see Materials and Methods for details). Moreover, an analysis of the fluorescence intensity histograms of individual heads that had packaged fluorescent DNA showed that the weighted average intensity for individual partial head samples was around 5,500 units (arbitrary units), while the same for proheads was 4,000 units, suggesting that the partial head packaged more DNA molecules than the prohead (Figure S3). This was further quantified by the number of photobleaching steps needed to bleach the fluorescent signal of each spot (Figure S4). These data showed that the partial head contained on average five to six DNA molecules per head, whereas the proheads and full heads contained four DNA molecules per head. Thus, the mature phage heads, like the procapsids, can undergo multiple packaging initiations [32],[33]. The single molecule data also suggest that the large difference in packaging efficiency between the partial head, the full head, and the prohead arose from the inability to initiate packaging in a large fraction of full heads and proheads. For heads that are capable of initiating DNA packaging, the number of molecules packaged is only slightly different between the three species.

## Discussion

One of the central themes in virus assembly is sequential and irreversible assembly. Assembly of one component generates a new site or conformational state that is specific for the assembly of the next component and so on [4],[25]. If a component is missing, assembly proceeds up to that point and stalls, accumulating a partially assembled structure and unassembled downstream components [15],[34]. Although the precise mechanisms are still poorly understood, the assembled structure does not spontaneously disassemble, nor is it in equilibrium with the unassembled subunits, presumably because it is locked in a different, energetically stable, conformational state. This process not only ensures directional assembly in a predetermined order but also leads to rapid and high-fidelity construction of a complex infectious virion from the seemingly chaotic distribution of subunits in the infected cell.

Consistent with the above paradigm, there is abundant evidence suggesting that sequential conformational changes in the portal and the major capsid protein drive maturation transitions from the nascent prohead to the DNA-full head [4],[5],[11]. These include assembly of the packaging motor, packaging initiation, prohead expansion, headful packaging, packaging termination, and assembly of neck proteins (Figure 1A–1D). The major capsid protein gp23* undergoes a major conformational change during prohead expansion, leading to a ∼15% increase in outer dimensions and a ∼50% increase in inner volume (gp23* is the cleaved form of the major capsid protein gp23; cleavage occurs during maturation of prehead to prohead; see Figure 1A). A conformational change in the T4 portal gp20 was reported to trigger this expansion following assembly of packaging motor on the unexpanded prohead [32]. About 870 binding sites for Soc (small outer capsid protein) and 155 binding sites for Hoc (highly antigenic outer capsid protein) are exposed following the expansion transition [35]. In phages SPP1 and P22, portal conformational variants were shown to either underpackage (∼95% of genome per head), or overpackage (∼105% of genome per head) [36],[37] the head. In phage P22, a piece of packaged DNA spools around the portal, forcing a conformational change that apparently signals the motor to make the headful termination cut and disengage from the DNA-full head [3]. Another portal conformational change primes DNA delivery following the binding of neck proteins [12]. Thus, the expectation was that the DNA-full heads, having just ejected the packaging motor following head filling, would not be competent to reinitiate packaging; instead, these would be primed to bind the neck proteins. Our results, however, show that the packaging machine assembly is neither sequential nor irreversible. It can occur on the finished head as efficiently as on the packaging-naïve empty (unexpanded or expanded) prohead, as was demonstrated by bulk as well as single molecule experiments. Such promiscuous assembly appears to be a special property of the packaging machine because all other head assembly transitions, for example head expansion, are irreversible and follow the classic sequential assembly paradigm. The fact that the motor can translocate DNA into the capsid regardless of its maturation state—unexpanded, expanded, DNA-full, or DNA-ejected—suggests that the shell as such is a passive receptacle. The main goal of the packaging process appears to be to power genome into a capsid receptacle until it is full.

What is the structural basis for the conformational plasticity of the packaging machine? X-ray and cryo-electron microscopy structures show that despite lacking sequence similarity, the three-dimensional structure of the portals is strictly conserved [7],[8]. The cone-shaped portal consists of three parts: a wide domain that is inside the icosahedral vertex, a long central stem that forms the channel, and a stalk that protrudes out of the capsid. The channel is lined by α-helices radiating from the center at a ∼45° angle, whereas the protruding end has an α/β domain connected by loops. In one model, the portal might oscillate between different energetically equivalent conformational states but gets “frozen” in one state upon binding to a partner molecule, gp17, gp13, etc. In another model, different binding sites may be accessible at different stages of the maturation pathway. In the nascent procapsid, only the protruding stalk would be accessed, allowing the assembly of gp17, but after head filling, the internal pressure of packaged DNA might push the portal down, exposing part of the stem that contains binding sites for neck proteins. Neck protein assembly displaces the packaging motor, but in the absence of neck proteins the packaging motor can reassemble to the portal.

A promiscuous packaging machine might have led to the evolution of headful genomes, a fundamentally common feature among dsDNA phages and viruses, including the herpes viruses [9]. Closed shells assembled from an ancient capsid protein probably predates genome evolution. A flexible packaging machine that can indiscriminately translocate DNA molecules into a capsid receptacle would continue packaging until the capsid is full. The filled shells, by virtue of the energy (internal pressure) present in the tightly packed DNA, can more efficiently deliver the “genome” into a host cell. Eventually, this selective advantage leads to the evolution of infectious capsids (virions) whose interior is tightly packed with DNA, their length dictated by the internal volume of the closed shell.

The conformational flexibility of the packaging machine may also lead to more efficient production of infectious virions in a normal infection. The low-abundant packaging/terminase proteins must compete for the DNA substrate with a variety of other DNA metabolizing enzymes involved in transcription, replication, recombination, and repair. Should the packaging motor prematurely fall off, or be displaced from the head, it could reassemble and resume packaging.

The use of highly stable virus shells as packaging containers is also a significant breakthrough from a technical standpoint and will have broad implications. First, the proheads currently used in all the in vitro DNA packaging systems are very fragile, and in T4 the prohead is a heterogeneous mixture of unexpanded, expanded, damaged, and partially Soc/Hoc-bound particles [27],[35]. The heads that have undergone all the maturation transitions are homogenous and structurally very stable and reinforced with 870 copies of Soc, offering a very efficient system to package DNA as well as generate high-resolution reconstructions of packaging intermediates. Furthermore, our results show that the partial heads have 5- to 10-fold greater packaging efficiency than the proheads. Second, it should now be possible to overcome some of the technical barriers to developing in vitro DNA packaging systems for eukaryotic viruses such as herpes viruses and adenoviruses by ejecting the packaged DNA from the virions [38] and repackaging different DNA into the emptied heads. Third, the powerful packaging motor can be used to encapsidate large chunks of foreign DNA and target these particles to specific cells or tissues by displaying specific ligands on the capsid surface [39]. Such particles can deliver multiple genes for gene therapy as well as multivalent DNA vaccines against pathogenic agents. The phage T4 head, which has very high capacity (∼170 kb) and is demonstrated to package multiple DNA molecules in the same head, would be a particularly attractive nanoparticle. Finally, it should be possible to design nanomotors for various biomedical applications. Since the shell appears to be a passive receptacle, the packaging machine (portal and motor) could be stripped off of the capsid and inserted into an artificial and much larger shell, such as a liposome or mammalian cell, and the machine could be made to translocate DNA and other therapeutic molecules into these compartments.

## Materials and Methods

### Purification of *10am13am* Heads

The phage heads, both partial heads and full heads, were isolated from the *Escherichia coli* P301 infected with *10am13am* mutant. Proheads were isolated from the *E. coli* infected with *17am18amrII* mutant. Proheads and phage heads were purified according to the procedures described earlier [30]. Briefly, the infected cells (500-ml culture) were lysed in 40 ml of Pi-Mg buffer (26 mM Na_2_HPO_4_, 68 mM NaCl, 22 mM KH_2_PO_4_, 1 mM MgSO4 [pH 7.5]) containing 10 µg/ml DNAse I and chloroform (1 ml) and incubated at 37°C for 30 min to digest DNA. The lysate was centrifuged at 4,300 *g* for 10 min, and the supernatant was centrifuged at 34,500 *g* for 45 min. The supernatant was resuspended in 2.5 ml of 50 mM Tris-HCl (pH 7.5) and 5 mM MgCl_2_, and again subjected to low- and high-speed centrifugations. The head pellet was then resuspended in 200 µl of Tris-Mg buffer and purified by CsCl density gradient centrifugation. The head bands (Figure 2A) were extracted and dialyzed overnight against 10 mM Tris-HCl (pH 7.5), 50 mM NaCl, and 5 mM MgCl_2_. The two closely spaced bands at the top were pooled and further purified by DEAE-Sepharose chromatography [30] (Figure 2B). The peak fractions were concentrated and stored at 4°C.

### Bulk In Vitro DNA Packaging

In vitro DNA packaging assays were performed by the procedure described earlier [30]. The reaction mixture contained purified proheads, partial heads, or full heads (0.5–1×10^10^ particles), purified full-length gp17 (1.5 µM), and DNA (300 ng of 50- to 766-bp ladder DNA [New England Biolabs], 100 ng of Cy3 83-bp DNA, 50 ng of Cy5 39-bp DNA, or 600 ng of 48.5-kb phage λ DNA). The λ DNA was packaged using a buffer containing 30 mM Tris-HCl (pH 7.5), 100 mM NaCl, 3 mM MgCl_2_, and 1 mM ATP. The Cy3 and Cy5 DNAs were packaged using the 5% PEG buffer as described earlier [30]. The packaging reactions were terminated by the addition of DNAse I, and the encapsidated DNAse I–resistant DNA was released by treatment with proteinase K and analyzed by agarose gel electrophoresis. Each experiment included one to several negative controls that lacked one of the essential packaging components: head, gp17, ATP, or DNA. Packaging efficiency is defined as the number of DNA molecules packaged per the number of head particles used in the packaging reaction.

### Single Molecule Optical Tweezers Packaging

The packaging complexes were prepared by mixing purified heads (4×10^9^ particles) with purified 1 µM full-length gp17 and 0.44 µM 125-bp dsDNA “priming” DNA (Z. Z. and V. B. R., unpublished data) in the presence of 1 mM ATP-γ-S in a 10-µl reaction volume consisting of packaging buffer (50 mM Tris-HCl [pH 7.6], 100 mM NaCl, and 5 mM MgCl_2_). The mixture was incubated at 37°C for 30 min. The T4-phage-antibody-coated polystyrene beads (1.5 µl) (0.79 µm in diameter, Spherotech) were added to the above reaction mixture and incubated at 37°C for 30 min.

The DNA beads were prepared by adding PCR-amplified 10-kb λ DNA biotinylated at one end to the streptavidin-coated polystyrene beads (0.86 µm in diameter, Spherotech) and incubated at 37°C for 30 min.

The dual-trap optical tweezers were set up and calibrated as described earlier [40],[41]. Single molecule measurements were taken at 100 Hz in a “force–feedback” mode, where packaging was allowed to occur against a constant force of 5 pN. Tether formation and packaging was initiated by infusing packaging buffer containing 1 mM ATP into the flow cell. To prevent the formation of reactive singlet oxygen species, an oxygen scavenging system was used (100 µg/ml glucose oxidase, 20 µg/ml catalase, and 4 mg/ml glucose). The contour length of DNA was calculated from the measured force and extension between the beads using the worm-like chain model assuming a persistence length of 53 nm, a stretch modulus of 1,200 pN and a distance per basepair of 0.34 nm. The velocity of DNA packaging was determined from a linear fit of the contour length of DNA over a sliding window of 0.1 s (ten data points).

### Single Molecule Fluorescence of Packaged Heads

Single molecule fluorescence experiments to quantify packaging efficiency of different heads were performed on a wide field prism-type total internal reflection microscope with a 532 laser (Coherent) for Cy3 excitation or a 630 laser (Melles Griot) for Cy5 excitation. Immobilized capsids were imaged by a charged-coupled-device camera (iXon DV 887-BI; Andor Technology) at 100-ms time resolution. A homemade C++ program was used to record and analyze the images [42].

To minimize nonspecific surface binding, clean quartz slides and glass cover slips were surface-passivated with PEG and 3% biotinylated PEG (Laysan Bio) [43]. After assembling the channel, NeutrAvidin (Thermo Scientific) was added (0.2 mg/ml), followed by incubation with biotinylated protein-G (Rockland Immunochemicals) (25 nM) for 30 min at room temperature. Subsequently, T4 phage antibody (15 nM) was added and incubated for 1 h. The packaged heads with 83-bp Cy3 and 39-bp Cy5 DNAs were applied to separate channels and incubated for 20 min. The packaging reaction mixtures were treated with DNAse I (10 µg/ml) at room temperature for about 20 h to digest any unpackaged or nonspecifically bound Cy3 and Cy5 DNAs. The unbound packaged heads were washed off, and immobilized capsids were imaged in 50 mM Tris-Cl buffer (pH 8), 5% PEG, 5 mM MgCl_2_, 1 mM spermidine, 1 mM putrescene, 60 mM NaCl, and an oxygen scavenger system (0.8% dextrose, 0.1 mg/ml glucose oxidase, 0.02 mg/ml catalase, and 3 mM Trolox) [44].

## Supporting Information

Figure S1 **Single molecule fluorescence of heads packaged with Cy5 39-bp DNA.** Representative images of partial heads, proheads, or full heads packaged with Cy5 39-bp DNA. The imaging area is 70 µm × 35 µm. Incubation time, laser intensity, imaging, and analysis parameters are the same for all samples.
(2.96 MB TIF)

Figure S2 **Single molecule fluorescence of heads packaged with Cy3 83-bp DNA.** Representative images of partial heads, proheads, and full heads packaged with Cy3 83-bp DNA. The imaging area is 70 µm × 35 µm. Incubation time, laser intensity, imaging, and analysis parameters are the same for all samples.
(2.33 MB TIF)

Figure S3 **Single head intensity for partial heads and proheads packaged with Cy3 83-bp DNA.** Normalized histograms showing single head intensity for partial heads and proheads. Intensity from more than 2,000 fluorescent particles was analyzed in each case. The intensity of partial heads was brighter than that of proheads. About 46% of imaged partial heads and only about 29% of proheads have intensity above 5,000, suggesting that the partial heads package more oligonucleotide molecules than the proheads (see Results for additional details).
(0.20 MB TIF)

Figure S4 **Photobleaching of a single packaged head.** Typical photobleaching steps from a single immobilized packaged head, packaged with multiple Cy5-labeled DNA fragments. Each step corresponds to one packaged labeled DNA.
(0.31 MB TIF)

## Abbreviations

DEAE: diethylaminoethyl cellulose
dsDNA: double-stranded DNA
PEG: polyethylene glycol
