# Supplementary figures and images for: A Promiscuous DNA Packaging Machine from Bacteriophage T4
Source: PLoS Biol. 2011 Feb 15;9(2):e1000592. doi: 10.1371/journal.pbio.1000592 (PMC3039672; doi:10.1371/journal.pbio.1000592)

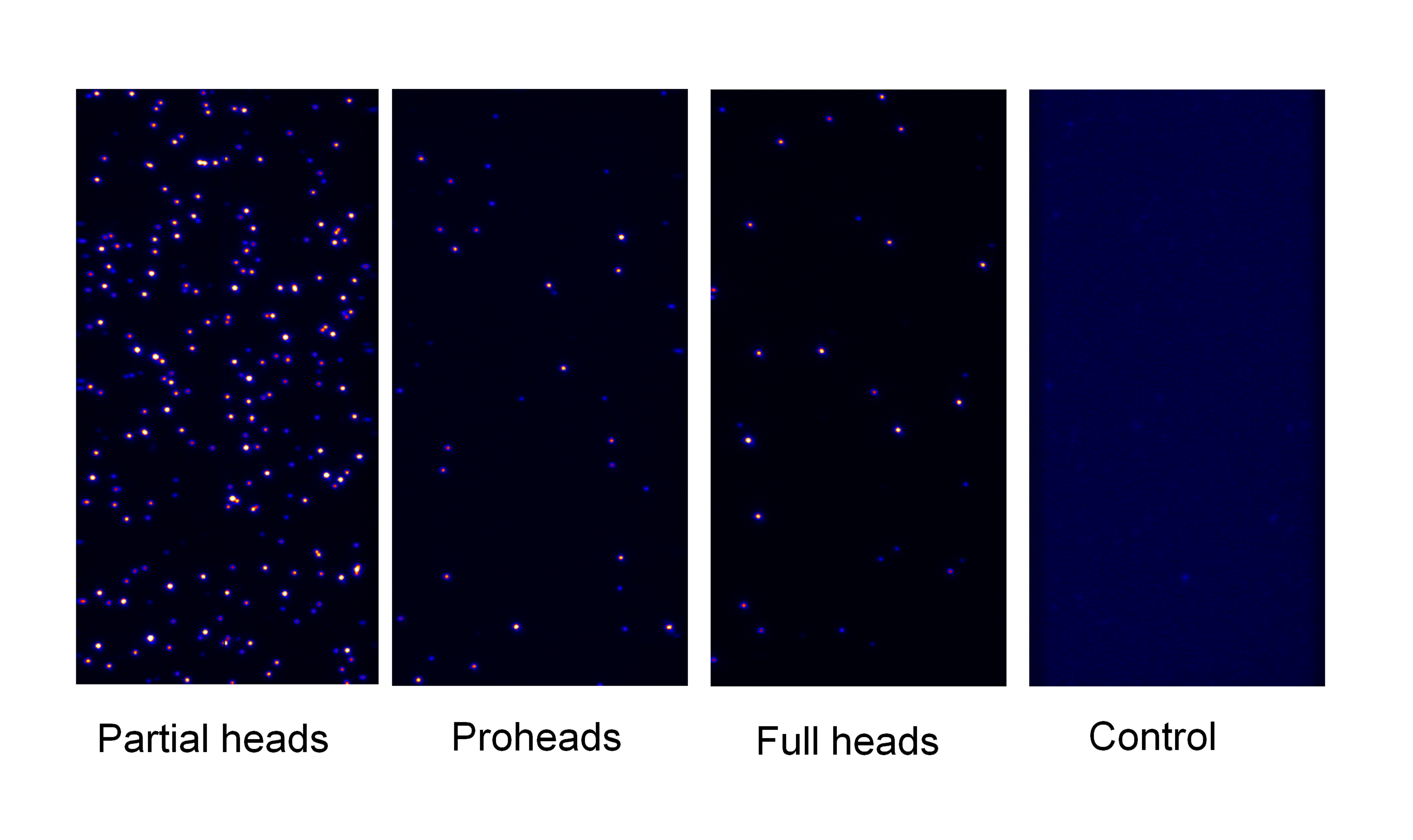

Supplement: Figure S1 — Single molecule fluorescence of heads packaged with Cy5 39-bp DNA. Representative images of partial heads, proheads, or full heads packaged with Cy5 39-bp DNA. The imaging area is 70 µm × 35 µm. Incubation time, laser intensity, imaging, and analysis parameters are the same for all samples. (2.96 MB TIF) [file pbio.1000592.s001.tif]

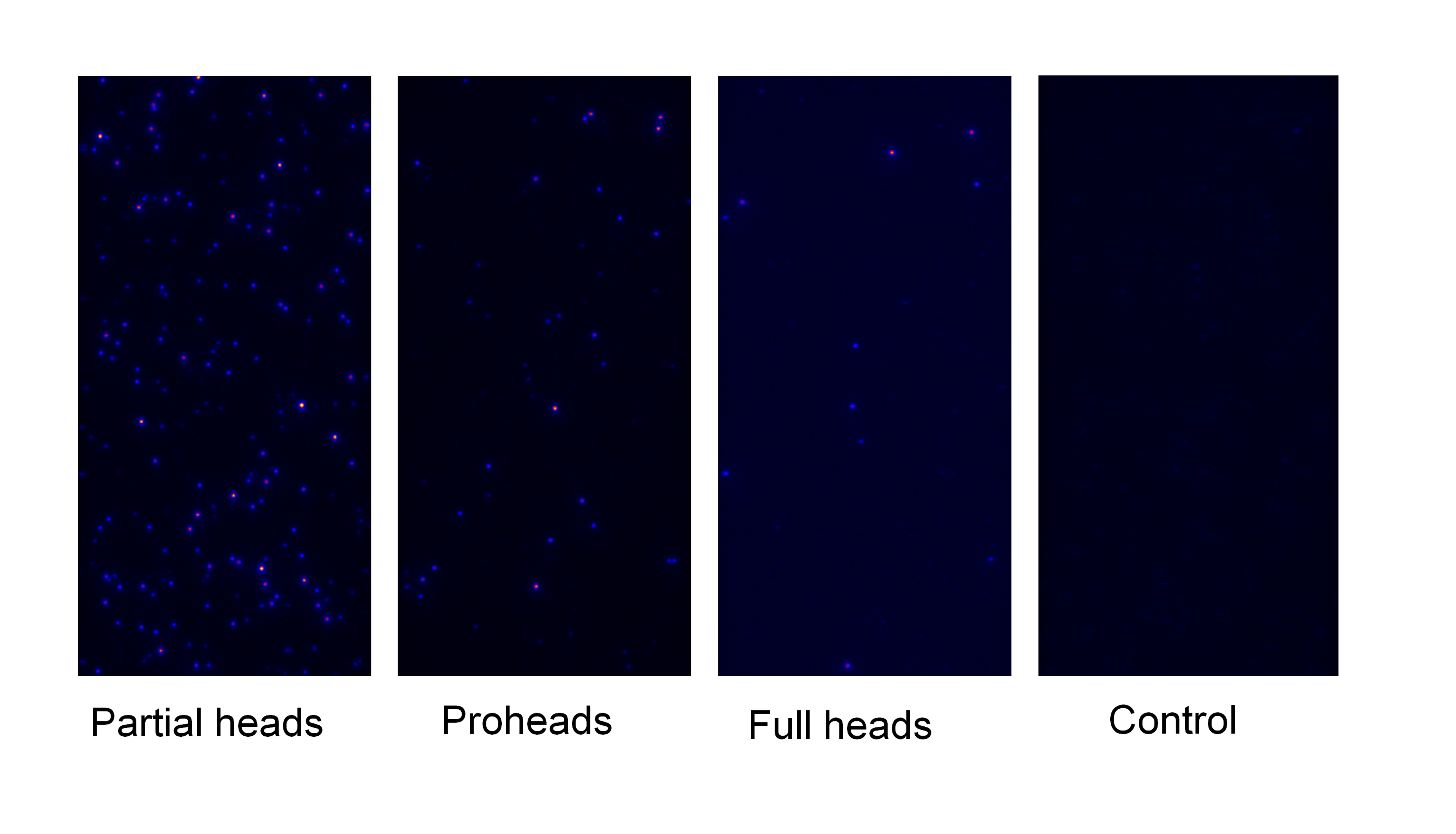

Supplement: Figure S2 — Single molecule fluorescence of heads packaged with Cy3 83-bp DNA. Representative images of partial heads, proheads, and full heads packaged with Cy3 83-bp DNA. The imaging area is 70 µm × 35 µm. Incubation time, laser intensity, imaging, and analysis parameters are the same for all samples. (2.33 MB TIF) [file pbio.1000592.s002.tif]

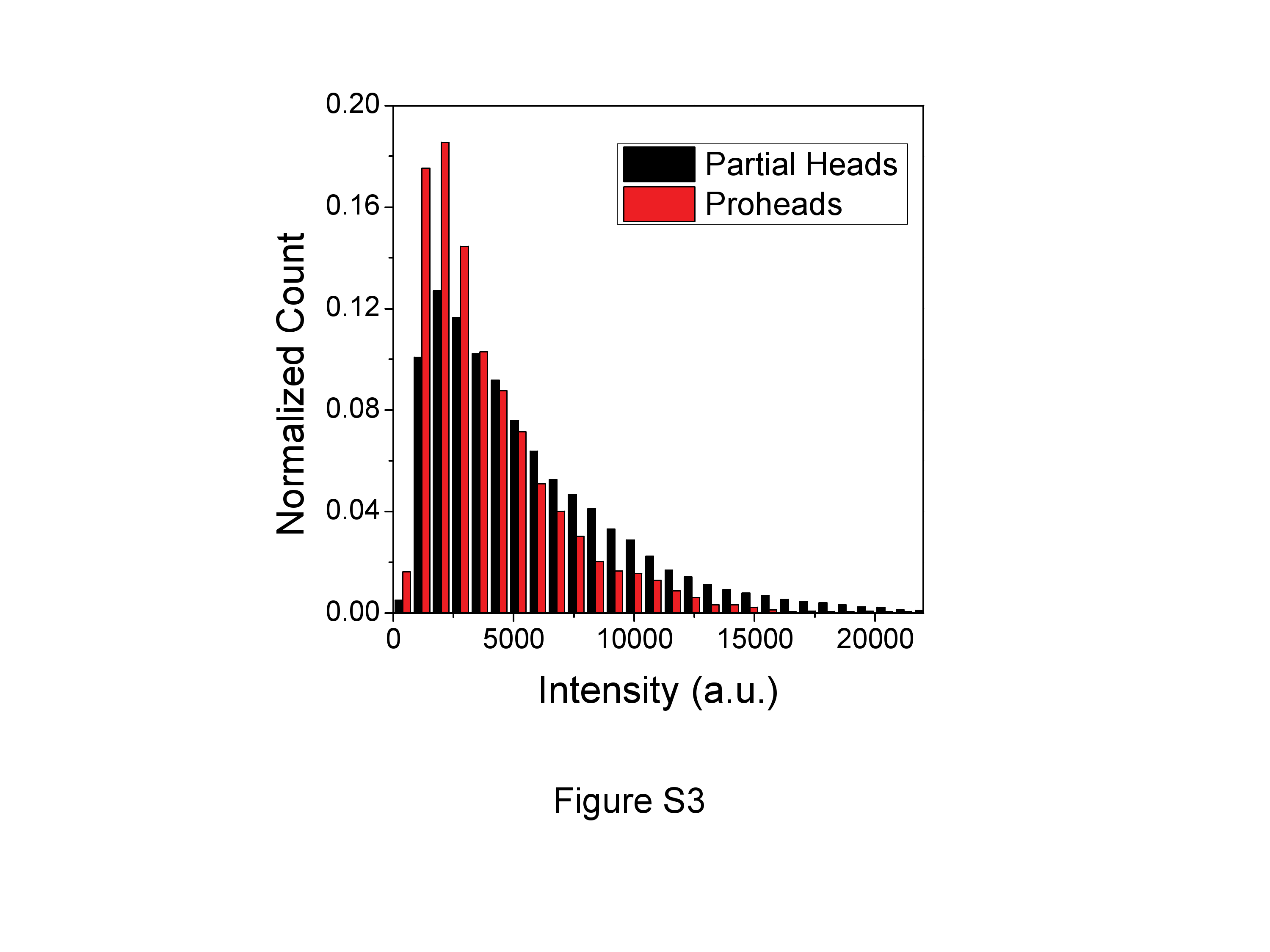

Supplement: Figure S3 — Single head intensity for partial heads and proheads packaged with Cy3 83-bp DNA. Normalized histograms showing single head intensity for partial heads and proheads. Intensity from more than 2,000 fluorescent particles was analyzed in each case. The intensity of partial heads was brighter than that of proheads. About 46% of imaged partial heads and only about 29% of proheads have intensity above 5,000, suggesting that the partial heads package more oligonucleotide molecules than the proheads (see Results for additional details). (0.20 MB TIF) [file pbio.1000592.s003.tif]

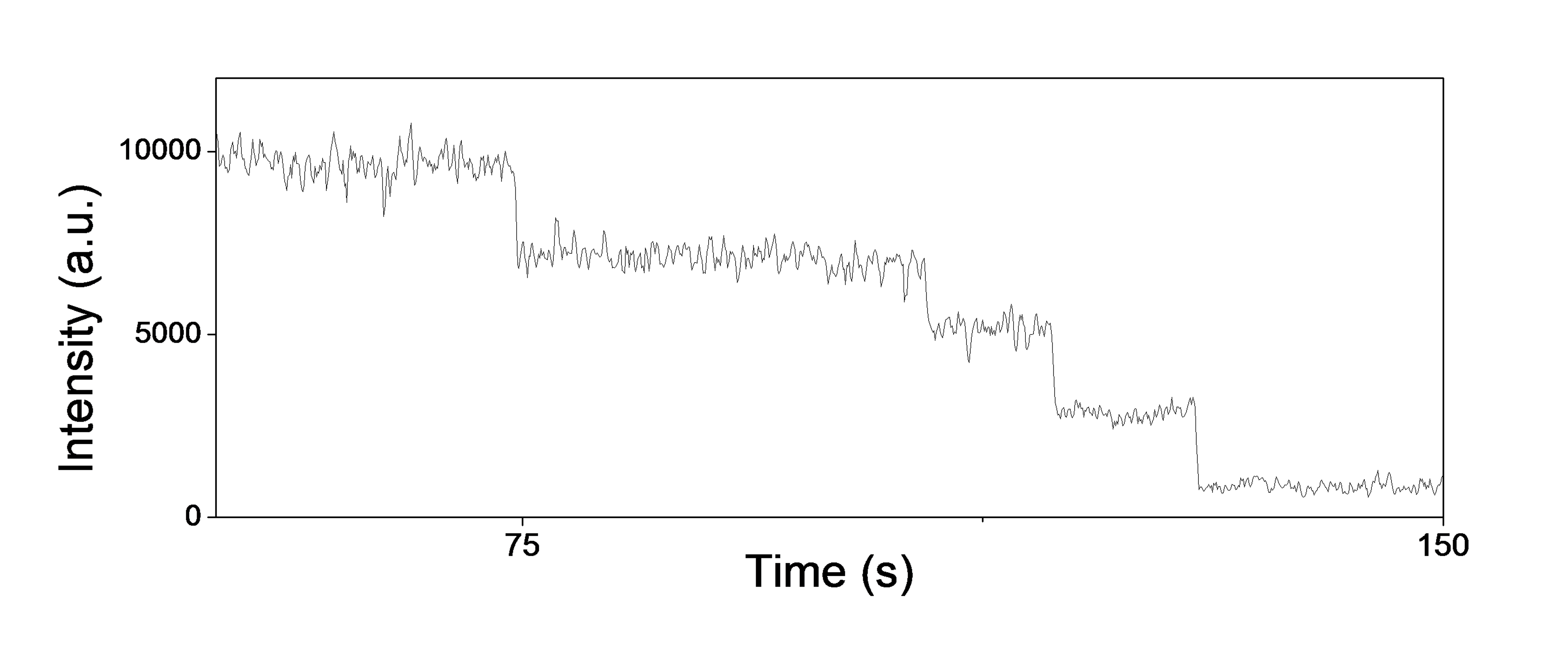

Supplement: Figure S4 — Photobleaching of a single packaged head. Typical photobleaching steps from a single immobilized packaged head, packaged with multiple Cy5-labeled DNA fragments. Each step corresponds to one packaged labeled DNA. (0.31 MB TIF) [file pbio.1000592.s004.tif]
